# Welding strength of NiTi wires

**DOI:** 10.1590/2177-6709.22.3.058-062.oar

**Published:** 2018

**Authors:** Tatyane Ribeiro Mesquita, Lídia Parsekian Martins, Renato Parsekian Martins

**Affiliations:** 1 Universidade Estadual Paulista, Faculdade de Odontologia de Araraquara, Departamento de Clínica Infantil (Araraquara/SP, Brazil).

**Keywords:** Welding, Orthodontic wire, Tensile strength

## Abstract

**Objective:**

To identify the appropriate power level for electric welding of three commercial brands of nickel-titanium (NiTi) wires.

**Methods:**

Ninety pairs of 0.018-in and 0.017 × 0.025-in NiTi wires were divided into three groups according to their manufacturers - GI (Orthometric, Marília, Brazil), GII (3M OralCare, St. Paul, CA) and GIII (GAC,York, PA) - and welded by electrical resistance. Each group was divided into subgroups of 5 pairs of wires, in which welding was done with different power levels. In GI and GII, power levels of 2.5, 3, 3.5, 4, 4.5 and 5 were used, while in GIII 2.5, 3, 3.5 and 4 were used (each unit of power of the welding machine representing 500W). The pairs of welded wires underwent a tensile strength test on an universal testing machine until rupture and the maximum forces were recorded. Analysis of variance (ANOVA) and *post-hoc* tests were conducted to determine which subgroup within each brand group had the greatest resistance to rupture.

**Results:**

The 2.5 power exhibited the lowest resistance to rupture in all groups (43.75N for GI, 28.41N for GII and 47.57N for GIII) while the 4.0 power provided the highest resistance in GI and GII (97.90N and 99.61N, respectively), while in GIII (79.28N) the highest resistance was achieved with a 3.5 power welding.

**Conclusions:**

The most appropriate power for welding varied for each brand, being 4.0 for Orthometric and 3M, and 3.5 for GAC NiTi wires.

## RESUMO

**Objetivo:**

identificar a potência mais adequada para solda elétrica em três marcas comerciais de fios de níquel-titânio (NiTi).

**Métodos:**

noventa pares de fios ortodônticos de NiTi, de dimensões 0,018” e 0,017” × 0,025’’, foram soldados por resistência elétrica e distribuídos em três grupos: GI (Orthometric, Marília, São Paulo, Brasil), GII (3M, São José do Rio Preto, São Paulo, Brasil) e GIII (GAC, Yorque, Pensilvânia, Estados Unidos). Cada grupo foi dividido em subgrupos de cinco pares de fios soldados com diferentes níveis de potência. Nos GI e GII, os fios foram soldados com níveis de potência de 2.5, 3, 3.5, 4, 4.5 e 5; enquanto no GIII as potências utilizadas foram de 2.5, 3, 3.5 e 4. Os pares de fios foram submetidos a um teste de tração até sua ruptura, com registro das forças máximas. Análises de variância (ANOVA) e testes *post-hoc* foram realizados para determinar qual subgrupo, dentro de cada grupo, obteve a maior força de resistência.

**Resultados:**

a potência 2.5 exibiu a menor resistência à ruptura para todos os grupos (43,75 N para o GI; 28,41 N para o GII e 47,57 N para o GIII). A potência 4 mostrou maior resistência à ruptura nos GI e GII (97,90 N e 99,61 N, respectivamente), enquanto no GIII (79,28 N) foi a potência 3.5.

**Conclusões:**

os níveis de potência mais adequados variaram para as marcas comerciais, sendo identificados como 4 para Orthometric e 3M, e 3.5 para GAC.

## INTRODUCTION

Electric resistance spot welding allows two or more metallic surfaces to be joined by the heat produced from an electric current conducted by two electrodes that hold both surfaces in tight contact.^1^ ^,^ ^2^ This procedure is routinely in orthodontics with stainless steel and beta-titanium wires, but has not been used with nickel-titanium (NiTi) alloys. The reason for that might be associated to orthodontic literature reports that NiTi wires cannot be welded,^3^ ^-^ ^6^ even though this information diverges from evidences on the field of materials engineering.^7^

NiTi wires could be further explored if welding is used. Short pieces of NiTi wires could be welded to the NiTi leveling wire, acting as hooks, allowing early use of elastics, and also as stops, decreasing cost in the orthodontic office. Moreover, springs made of NiTi could also be welded to the main wires, allowing use of superelasticity, shape-memory and low deformation of NiTi in nonconventional clinical situations. Thus, an approximate weld of round wires, usually used as initial leveling wires, to rectangular wires might he used for the mentioned situations. 

Even though four papers on electrical resistance welding of NiTi wires have been published,^8^ ^-^ ^11^ two of them did not compare the resistance to rupture among the welding power used.^8^ ^,^ ^9^ Moreover, none of them tested the welding configuration of round to rectangular wires and did not use more than one commercial brand of NiTi. If assumed that different wires might exhibit different responses to heat, incorrect power levels used for welding may damage the wires. 

Therefore, the present study aims to test the weld resistance of NiTi wires, in order to identify the most appropriate power level to be used in wires manufactured by three different trademarks. 

## MATERIALS AND METHODS

Ninety pairs of round 0.018-in and 0.017 × 0.025-in NiTi wires were divided into three groups according to their manufacturers: Group I (GI) was made with wires from Orthometric (Marília, São Paulo, Brazil), Group II (GII), from 3M (3M OralCare, St. Paul, CA); and Group III (GIII), from GAC International (GAC, York, PA, United States). 

**Figure 1:**
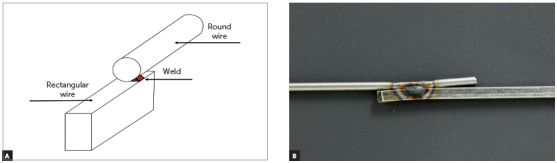
A) Schematic drawings of the electric resistance weld. B) Image of a weld analyzed in this study.

The thinner surface of the rectangular wire of each group was welded by electrical resistance to a respective round wire (Fig 1), using a modified spot welding machine (Pontomatic NiTi - Electronic Automatic, Kernit, Indaiatuba, São Paulo, Brazil). Subgroups were created within each group, according to the power levels used for the welding. Groups I and II were divided into six subgroups, which were welded with power levels of 2.5, 3, 3.5, 4, 4.5 and 5 (each power level of the welding machine represents 500W), while Group III wires were divided into four subgroups, welded with power levels of 2.5, 3, 3.5 and 4. The tests started at a power level of 2.5 because this was the smallest power capable of welding wires, while higher values than the ones tested damaged the wires during welding. The welding machine was calibrated to apply an electric current for 3 milliseconds between two flat electrodes, producing a force of 12N when joining the wires. All the welding procedures were performed by the same calibrated operator. 

For the tension test, the welded wires were secured at a distance of 4 cm from the weld, with clamps specially made for the study, and subjected to the tensile strength tests using a universal testing machine (EMIC, São José dos Pinhais, Paraná, Brazil), with a 5 kN load cell. The tests were performed with a crosshead speed of 0.5 mm per minute until the rupture of the wires. The dedicated software of the machine recorded the breaking-point force, in Newtons (N). 

Statistical analysis was performed using SPSS software, version 16.0. (SPSS Inc., Chicago, IL, USA). The data distribution was normal and subgroups were compared using an analysis of variance with a significance level of 0.05, to identify subgroup differences while Tukey *post-hoc* test (*p*= 0,05) was used to determine which groups were different. 

## RESULTS

**Figure 2:**
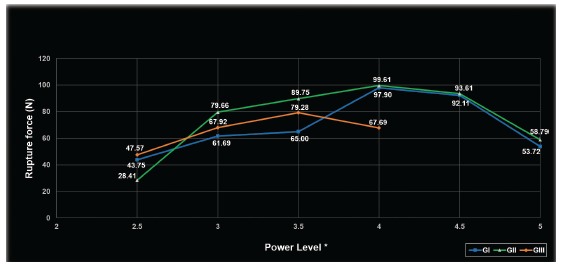
Graph of maximum tensile strength of the welds performed in NiTi wires of the tested groups, according to the used power levels. * One unit of power level of the welding machine represents 500W.

All the wire ruptures were very close to the weld. There was a difference between the forces registered in the GI subgroups (*p*< 0.001) (Table 1). The power levels of 2.5, 3, 3.5 and 5 produced similar forces of rupture, with values of 43.75N, 61.69N, 65.00N and 53.76N, respectively. The power levels 4 and 4.5 were statistically similar to each other, but different from the remaining levels, with forces of 97.90N and 92.11N, respectively (Fig 2). 

**Table 1:** Average and standard deviations of the tensile strength (in Newtons) of NiTi wires welded at different power levels.

Power level	GI (N)	GIII (N)	GIII (N)
2.5	43.75 ± 6.10 ^a^	28.41 ± 12.36 ^a^	47.57 ± 4.39 ^a^
3	61.69 ± 9.41 ^a^	79.66 ± 7.64 ^b.c^	67.96 ± 4.77 ^a.b^
3.5	64.99 ± 11.11 ^a^	89.75 ± 12.48 ^c^	79.28 ± 12.62 ^b^
4	97.90 ± 11.59 ^b^	99.61 ± 10.14 ^c^	67.69 ± 18.14 ^a.b^
4.5	92.11 ± 17.51 ^b^	93.61 ± 15.74 ^c^	...
5	53.74 ± 14.39 ^a^	58.79 ± 18.29 ^b^	...
Sig.	<.001	<.001	.004

Each unit of power level of the welding machine represents 500W. Equal letters: absence of statistically significant difference (*p* > 0.05, ANOVA).

There was also a difference between the force values of GII (*p*< 0.001). The power level 2.5 resulted in a maximum force (28.41N) different to the other power levels. The power levels 5 and 3 (58.79N and 79.66N, respectively) produced similar forces, which were different from the remaining levels. The levels 3, 3.5, 4 and 4.5 were statistically similar and produced forces of 79.66N, 89.75N, 99.61N and 93.61N, respectively (Fig 2). 

GIII also showed a difference between the registered forces (*p*= 0.004). The weld with a power level of 2.5 produced a maximum force of 47.57N, which was similar to the power levels 3 (67.96N) and 4 (67.69N). The power level 3.5 produced a maximum force of 79.28N, which was statistically similar to the power levels 3 and 4. The power levels 4.5 and 5 were not tested as the wires were destroyed during welding (Fig 2). 

## DISCUSSION

The power that produced the strongest weld in the NiTi wires was identified for each tested brands. Although there are only four articles in the literature related to electric resistance welding in NiTi wires,^8^ ^-^ ^11^ none of them tested wires of different commercial brands, which is very important, according to the present results. Moreover, none of them tested which power was more appropriate to be used according to the resistance of the weld. 

The most appropriate power level for each brand was defined as the one that produced a weld with the highest tensile strength. The tests started at a power level of 2.5 - since lower levels did not weld the NiTi wires -, and these power levels were increased in steps of 0.5 until the heat produced by the weld broke the wires during the welding process. Present results show that the maximum tensile strength of the welded wires increased with the power levels up to a certain point, after which the resistance began to decrease. These peaks of resistance (Fig 2) were at the power 4 for the GI and GII groups, and 3.5 for GIII group. The resistance decrease with higher power levels occurred because the heat produced by the weld causes annealing of the wire^10^, which is sufficient to significantly alter its mechanical properties and sometimes even break the wire, as occurred in the wires of the GIII group when welded at power levels higher than 4 (and therefore were not used in this study). The difference of power levels that obtained the highest resistance for each group can be explained by variations in the manufacturing process of the wires, which may be due to its composition, as the nickel percentage can vary,^12^ or due to the tested welding machine setting and cooking processes. 

The rupture of the wires never occurred at the weld but very close to it. This was probably due to the heat produced around the weld, which anneals the wire making it less resistant. Welded wires show less maximum resistance to tension than solid wires,^8^ ^,^ ^13^ ^-^ ^15^ however, it need to be strong enough to tolerate orthodontic forces. In this report, it were found values ranging from 8 to 10 kilograms-force, which are high enough to support orthodontic forces. Even though the objective of this paper was not to compare the tensile forces of NiTi and stainless steel (SS) wires, a tension test was performed on same dimension SS wires (3M OralCare, St. Paul, CA) for discussion purposes. This comparison was not present in the objectives of this study, since SS wires need a higher power to produce adequate weld than NiTi wires, due to its higher melting point. Nonetheless, necessarily using a higher power level (5), the SS wires showed an average force of resistance to rupture of 134.3 ± 6.96 N, which was approximately 35, 37 and 69% higher than for the GII, GI and GIII groups, respectively. 

**Figure 3:**
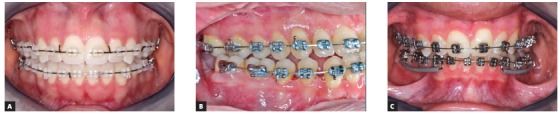
A) Welded NiTi hooks for vertical elastic use. B) Welded stop for tying the archwire. C) Stops to keep wire from sliding distally.

**Figure 4:**
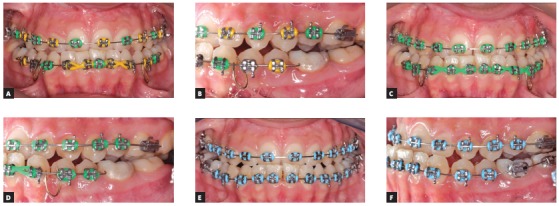
0.014-in NiTi proclining loops welded to the main wire, initial stage: A) frontal view and B) lateral view. Same patient, 30 days after: C) frontal view and D) lateral view. Same patient, after 30 more days: E) frontal view and F) lateral view.

This results are lower than the results reported in the literature^8^ ^-^ ^10^ because the wires used in this work had smaller diameters. The clinical implications of welded NiTi orthodontic wires are of great importance as they allow a combination of the advantages of this alloy, such as high flexibility, shape memory, superelasticity, the possibility of using hooks for attaching elastics, and the use of omega loops or stops (Fig 3). Additionally, springs may also be welded to alignment wires, to assist in that phase of treatment (Fig 4). 

Although these results open up new possibilities for the use of NiTi alloys in orthodontics, further studies should be made to evaluate these welded wires in the long term within the oral cavity, where they are subjected to factors such as corrosion by saliva as well as thermal and mechanical cycling. Additionally, it would be of great value to develop, if possible, a type of weld in which there is no annealing of the wire around the weld point, both for NiTi wires and steel wires, which are usually reinforced with a silver alloy weld. 

## CONCLUSIONS

The most suitable power levels for electric resistance welding of NiTi wires varied for the different brands. 

The most appropriate power level to weld the wires with the parameters given by the used welding machine was identified as being 4 (2000W) for the Orthometric and 3M brands, and 3.5 (1750W) for GAC.

## References

[1] Darwish S, Ghanya A (2000). Critical assessment of weld-bonded technologies. J Mater Process Technol.

[2] Wu K (1975). Resistance spot welding of high contact resistance for weld-bonding. Weld J.

[3] Kapila S, Sachdeva R (1989). Mechanical properties and clinical applications of orthodontic wires. Am J Orthod Dentofacial Orthop.

[4] Iijima M, Brantley WA, Yuasa T, Muguruma T, Kawashima I, Mizoguchi I (2008). Joining characteristics of orthodontic wires with laser welding. J Biomed Mater Res B Appl Biomater.

[5] Blackman R, Baez R, Barghi N (1992). Marginal accuracy and geometry of cast titanium copings. J Prosthet Dent.

[6] Burstone CJ (1987). Welding of TMA wire. Clinical applications. J Clin Orthod.

[7] Duerig P, Collings EW, Welsch G (1994). TiNi Shape Memory Alloys. Material Properties Handbook Titanium Alloys..

[8] Matsunaga J, Watanabe I, Nakao N, Watanabe E, Elshahawy W, Yoshida N (2015). Joining characteristics of titanium-based orthodontic wires connected by laser and electrical welding methods. J Mater Sci Mater Med.

[9] Nascimento LE Santos RL, Pithon MM Araújo MT, Nojima MG LI. N (2012). The effect of electric spot-welding on the mechanical properties of different orthodontic wire alloys. Mat Res.

[10] Tam B, Pequegnat A, Khan MI, Zhou Y (2012). Resistance Microwelding of Ti-55 8 wt pct Ni Nitinol Wires and the Effects of Pseudoelasticity. Metall Mater Trans A.

[11] Fukumoto S, Morikawa SAY (2008). Small-scale resistance welding of crossed TiNi fine wire. Mater Sci Forum.

[12] Kararia V, Jain P, Chaudhary S, Kararia N (2015). Estimation of changes in nickel and chromium content in nickel-titanium and stainless steel orthodontic wires used during orthodontic treatment an analytical and scanning electron microscopic study. Contemp Clin Dent.

[13] Sevilla P, Martorell F, Libenson C, Planell JA, Gil FJ (2008). Laser welding of NiTi orthodontic archwires for selective force application. J Mater Sci Mater Med.

[14] Bock JJ, Fraenzel W, Bailly J, Gernhardt CR, Fuhrmann RA (2008). Influence of different brazing and welding methods on tensile strength and microhardness of orthodontic stainless steel wire. Eur J Orthod.

[15] Gugel H, Schuermann A, Theisen W (2008). Laser welding of NiTi wires. Mater Sci Eng A.

